# *Fusarium* Species and *Fusarium oxysporum* Species Complex Genotypes Associated With Yam Wilt in South-Central China

**DOI:** 10.3389/fmicb.2020.01964

**Published:** 2020-08-17

**Authors:** Fang Dongzhen, Liu Xilin, Chen Xiaorong, Yan Wenwu, He Yunlu, Cheng Yi, Chen Jia, Li Zhimin, Guo Litao, Wang Tuhong, Jianping Xu, Gao Chunsheng

**Affiliations:** ^1^Institute of Bast Fiber Crops and Center of Southern Economic Crops, Chinese Academy of Agricultural Sciences, Changsha, China; ^2^Key Laboratory of the Biology and Processing of Bast Fiber Crops, Ministry of Agriculture, Changsha, China; ^3^Yichun Agricultural Science Research Institute, Yichun, China; ^4^Department of Biology, McMaster University, Hamilton, ON, Canada

**Keywords:** yam (*Dioscorea* L.), yam wilt, host-pathogen association, multiple infections, multilocus microsatellite genotyping, gene flow, geographic differentiation

## Abstract

Chinese yam (*Dioscorea polystachya* Thunb.) is an important root crop. Wilt caused by *Fusarium* is among the most important emerging diseases on yams. However, there is currently limited information on the molecular epidemiology of *Fusarium* causing yam wilt. Here, we investigated wilted yam samples from six regions in South-Central China. A total of 117 *Fusarium* isolates were obtained from diseased tissues of 37 wilted yam plants. These yam plants belonged to two varieties characterized by white and purple fleshy tubers, respectively. Analyses of *ef1-α* sequences identified that these 117 *Fusarium* isolates belonged to 11 putative species, with *F. aff. commune* being the most common (31.6%), followed by *F. aff. cugenangense* (29.1%), a potential undescribed species *Fusarium aff*. sp. (11.1%), *F. aff. gossypinum* (9.4%), *F. aff. fujikuroi* (8.5%), *F. aff. nirenbergiae* (6%), and one isolate each (0.85%) of *F. aff. asiaticum*, *F. aff. curvatum*, *F. aff. odoratissimum, F. aff. solani*, and *F. aff. verticillioides*. Six of these species were recently described as new species within the *Fusarium oxysporum* species complex (FOSC). Interestingly, 18 of the 37 yam plants were infected by two or more *Fusarium* species each and there was evidence for differential *Fusarium* species distributions based on geographic location and/or yam host variety. Multilocus microsatellite genotyping of the 67 FOSC isolates revealed that isolates of the same species from the same diseased plants often belonged to different genotypes. Interestingly, several FOSC microsatellite genotypes were shared among distinct geographic regions, consistent with long-distance dispersal. However, population genetic analyses revealed significant contributions of geographic separation to the overall genetic variation of FOSC with several pairs of geographic populations showing significant genetic differentiations, consistent with differential geographic distribution of the species within FOSC. The implications of our results to the managements of *Fusarium* wilt in yams were discussed.

## Introduction

The filamentous fungal genus *Fusarium* has a worldwide distribution and contains at least 300 phylogenetically distinct species/species complexes (O’Donnell et al., 2015). This ascomycete genus is among the world’s most economically destructive plant pathogens, capable of causing diseases of almost all economically important plants and resulting in damages worth billions of dollars to the global agriculture each year (Aoki et al., 2014). Some of the *Fusarium* species are also opportunistic human pathogens, causing infections of cornea and nails, etc. In addition, *Fusarium* fungi can produce a diversity of toxic secondary metabolites, such as trichothecenes, zearalenone, and fumonisins, posing significant threats to food safety and human health (Marasas et al., 1984). Consequently, there have been sustained interests among mycologists, plant pathologists, and food specialists to develop approaches and methods to control *Fusarium* pathogens. Understanding the distribution and patterns of spread of *Fusarium* fungi represents a critical step in developing such strategies. One important group of crop plants that suffer from *Fusarium* infections are the yams (Yao, 1998). However, at present, our understanding of the epidemiology of *Fusarium* pathogens on yams is very limited.

Yams are a group of economically important, annual or perennial plants, capable of producing underground tubers. They provide a major source of staple food for 60–100 million people in Africa, South America, the Pacific and Asia (Price et al., 2018). Globally, cultivated yams are ranked as the fourth most important root crop by production, after potato, cassava and sweet potato (Sukal et al., 2017). Yams are typically dioecious, monocotyledonous, with vines extending over several meters or more (Mignouna et al., 2008). The common yams are members of *Dioscorea* L, a large plant genus containing about 600 species. Yams are broadly cultivated in tropical and subtropical regions in Southeast Asia, Africa, Central America, and South America, spanning the entire globe along the so-called “yam belt” (Huber, 1998; Andres et al., 2017). Common types of cultivated yams include white yam (*D. rotundata*), yellow yam (*D. cayenensis*), water yam (*D. alata*), air potato (*D. bulbifera*), bitter yam (*D. dumetorum*), lesser yam (*D. esculenta*), and Chinese yam (*D. polystachya*).

The Chinese yam is commonly known as Shanyao in Chinese (*D. batatas, D. opposite*, and *D. polystachya*). This species is native to China and has been artificially cultivated for more than 1000 years (Peng et al., 2017). It is commonly found in valleys and on the sunny side of hills across China, especially in southern regions. The plant is a perennial, smaller than the African yams but capable of growing over 3 meters tall and 1.5 meters wide. However, its tolerance to low temperatures has enabled its cultivation in much cooler climates such as northern China, Korea, and Japan. Among regions within China, the long domestication and cultivation history of *D. polystachya* have resulted in a variety of local cultivars, including Ma Yam, Ruichang Yam, and Foot Potato (Gao and Zhao, 2007). These varieties are not only a common food but also used as a traditional Chinese medicine by locals for the treatment of diabetes, diarrhea, asthma, and other ailments (Ju et al., 2014; Chen et al., 2015).

*Fusarium* is among the most common disease agents of yams, causing wilts, rots, and damping-off diseases (Srinivas et al., 2019). *Fusarium* pathogens can infect both the above-ground and below-ground parts of yams starting at the early stage of yam development, with infected tissues continuously change color, resulting in vascular wilt, and eventually rot and plant death (Zhu et al., 2016). The disease can continue its spread even after harvesting, during storage of yams. It is estimated that *Fusarium* infections may cause 30∼70% of yam crop reductions each year in China (Sun et al., 2012). The yam wilt disease was first reported in China in 1988, and the initial disease agent was identified as *Fusarium oxysporum* (Yao, 1998). Since then, yam wilt disease has been reported from many regions in China, including Hebei (Liu et al., 2019), Henan (Shao and Li, 2019), Hainan (Huang et al., 2013; Wu L. et al., 2019), and Jiangxi (Tu et al., 2012) provinces. So far, these studies have investigated relatively few isolates and with morphological characters as the main criteria for pathogen species identification. All studies reported *F. oxysporum* as the dominant pathogen but with intra-specific genetic variation unknown. Furthermore, morphological features are often insufficient for the identification of *Fusarium* species, especially for distinguishing closely related species. Instead, molecular markers such as DNA sequences are increasingly used for species identification. Indeed, the use of DNA sequences from multiple loci have resulted in the discovery of many new species within previously described species. For example, a recent study revealed 15 new species within the *Fusarium oxysporum* species complex (FOSC), plus several new ones still to be described (Lombard et al., 2019). In addition, molecular markers such as simple sequence repeats (SSR) have been increasingly used for identifying fungal pathogen genotypes, such as for strains within FOSC (Bogale et al., 2005; Cruz et al., 2018). SSR markers are based on copy number variations of one or a few nucleotide repeats within specific genomic regions. The changing taxonomy of *Fusarium* species requires that our pathogen identification criteria need to be updated. Furthermore, the availability of genetic markers such as SSR enables genotyping of *Fusarium* strains and inferences of molecular epidemiology of *Fusarium* causing yam wilt.

According to climatic conditions and the characteristics of yam production, China can be divided into five main yam cultivation areas: Northeastern, North-Central, South-Central, Far Southern, and Northwestern Arid Areas (Gao and Zhao, 2007). Located in South-Central China, Jiangxi province is one of the main yam-producing regions, with an estimated cultivation area of 5200 hm^2^ (Yao et al., 2018). Yams are planted in various parts of Jiangxi province, and different areas often have their own local varieties, developed through their long histories of cultivation and selection. In these areas, while some farmers rotate their crops, due to the high yield and increasing profit for growing yams, most farmers nowadays tend to grow yams in the same fields year after year, especially during the last 30 years. Coincidentally, over the last few decades, there has been increasing trend of wilt diseases of yams in Jiangxi, leading to serious economic losses (Huang et al., 2014). However, the underlying pathogen(s) and the epidemiology are unknown. The objectives of this study are to identify the *Fusarium* species and strains causing yam wilt and investigate the relationships among pathogen strains and populations.

## Materials and Methods

### Fungal Isolation

Underground parts (including roots, basal stems, and tubers) of yam plants with wilt symptoms were collected from six regions in two neighbor provinces in South-Central China, Jiangxi, and Hunan provinces. Five of the regions were located in Jiangxi province: Yichun, Ruichang, Ji’an, Ganzhou, and Fuzhou. The sixth geographic region was in Yiyang in Hunan province (Table 1). The geographic coordinates of the sampled sites are presented in Table 1. Based on the color of the tuber flesh, the yams analyzed here belonged to two cultivar types, corresponding to white and purple fleshy tubers. All diseased plant samples were collected from May to August 2018. Three of the geographic regions (Ruichang, Fuzhou, and Yiyang) had only the white colored variety. One region (Ganzhou) had only the purple colored variety. The remaining two geographic regions (Yichun and Ji’an) had a mixture of both yam varieties, with Yichun dominated by the purple variety while Ji’an dominated by the white variety.

**TABLE 1:** Sampling locations, sample sizes and diversity of *Fusarium* species causing yam wilt in Jiangxi and Hunan provinces, China.

Province	Local site	Geographic coordinates	Number of wilted plants investigated	Number of plants with *Fusarium*	Number of putative *Fusarium* species (strains)	*Fusarium* species diversity
Jiangxi	Ruichang	115.63E 29.67N	11	8	5 (30)	0.73
Jiangxi	Yichun	114.45E 27.79N	15	9	4 (30)	0.69
Jiangxi	Ji’an	115.42E 27.31N	9	8	7 (28)	0.70
Jiangxi	Ganzhou	114.62E 25.66N	6	4	3 (9)	0.57
Jiangxi	Fuzhou	116.36E 27.95N	2	2	3 (5)	0.56
Hunan	Yiyang	112.21E 28.60N	9	6	4 (15)	0.52
Total	−	−	52	37	6 (117)	−

Fungal pathogens from the diseased tissues were isolated following the procedures described below (Fang, 1998). Briefly, the below-ground tissues were washed with tap water to remove soil debris. The tissues were then surface-sterilized in 75% ethanol (V/V) for 30s, followed by submerging in 1% NaOCl (W/V) for 10s, and rinsed three times in sterile distilled water. The diseased parts were then cut to smaller pieces (approximately 1 cm^2^) and placed on 9-cm-diameter polystyrene Petri dishes containing potato dextrose agar (PDA, 200 g L^–1^ potato, 10 g L^–1^ glucose, and 15 g L^–1^ agar) supplemented with 50 μg ml^–1^ streptomycin. The main tissues analyzed here were the diseased below-ground stem at the junction between the tuber and the above-ground stem. However, in situations where the tubers were severely infected, diseased tubers were also treated, cut, and placed on the medium for pathogen isolation. The Petri dishes were incubated at 25°C for 7–10 days in the dark. Pure fungal isolates were obtained by either excising a hyphal tip from the colonial margin or by a single-spore isolation method (Zhou et al., 2015). Fungal culture from each diseased tissue represents an independent isolate. For certain plants, multiple independent diseased tissues were incubated and multiple fungal strains were obtained from each diseased plant. The isolated strains were first identified morphologically using a compound microscope. The fungal isolates with characteristic colony and microscopic morphological features of the genus *Fusarium* were then transferred onto new PDA dishes for further DNA extraction and genotyping analysis.

### DNA Extraction, PCR Amplification, and Phylogenetic Analysis

From 7-day-old isolates cultured on PDA, the mycelia were collected and then frozen in liquid nitrogen and ground with Tissuelyer-24 full-automatic sample grinder (Shanghai Jingxin Industrial Development Co., Ltd., China). Total genomic DNA was extracted from each isolate using a SP Fungal DNA Kit (Aidlab Biotech, Beijing, China) according to the manufacturer’s instructions. To confirm the species identity of the isolated *Fusarium* pathogens, we followed the recommendations by O’Donnell et al. (2015) and obtained the DNA sequence at the translation elongation factor α (*ef1*-α) gene from each isolate (Chang et al., 2015). Briefly, PCR amplification was conducted in a final volume of 25 μL containing 1 μL of genomic DNA, 1 μL of each primer (10 μM), 12.5 μL of Taq PCR MasterMix (Sangon Biotech, Shanghai, China). The *ef1*-α region was amplified with the primer pair EF1 (ATGGGTAAGGARGACAAGAC) and EF2 (GGARGTACCAGTSATCATG) (O’Donnell et al., 2015). PCR amplification was performed in a T100^TM^ Thermal Cycler (Bio-Rad Laboratories) with an initial denaturation at 94°C for 5 min, 35 cycles of amplification and a final extension at 72°C for 10 min; each cycle of amplification consisted of denaturation at 95°C for 30 s, annealing at 55°C for 30 s, and extension at 72°C for 1 min. Successful PCR amplifications were confirmed by agarose gel electrophoresis (1 × agarose in TBE buffer). Amplified PCR products were then purified and sequenced by TSINGKE Biological Technology Company (Changsha, China).

For phylogenetic analysis, representative *ef1*-α sequences of *Fusarium* species closely related to our sequences were retrieved from GenBank. Whenever possible, sequences from type and epitype strains were retrieved for analyses, including the recently described new species within FOSC (Lombard et al., 2019). These sequences were then combined with our own sequences for phylogenetic analyses and to identify the most likely species affiliation of our isolates. Phylogenetic analysis was conducted with the MEGA 7.0 software using the Maximum Likelihood (ML) algorithm (Kumar et al., 2016). Clade support was inferred from 1000 bootstrap replicates, and alignment gaps were excluded.

### Pathogenicity Tests of Representative Isolates of *Fusarium* Species

To confirm that the obtained *Fusarium* isolates were capable of causing disease in Chinese yams, we conducted pathogenicity tests for representative pathogen isolates from two regions and tested those isolates on their local yam hosts. The two regions were Ji’an and Yichun where we were able to obtain fresh plants for the white yam and purple yam, respectively this season (June−July 2020) for testing. Specifically, one random isolate from each of four *Fusarium* species isolated from white yam in Ji’an was tested on white yam plants. Similarly, one random isolate from each of four *Fusarium* species isolated from purple yam in Yichun was tested on purple yam plants. Due to the long growth period of yams, seasonality of the yam plants, and the lack of a standard method for producing wilt in yams, the pathogenicity tests were conducted using yam leaves as described earlier (Huang et al., 2013; Han et al., 2019).

To test the pathogenicity of each *Fusarium* isolate, seven healthy leaves of the same size from the middle of yam vine were taken and cleaned with tap water. The leaf surface was disinfected with 75% ethanol for 30s, then rinsed with sterile water. After air dry, each leaf was put in a sterile glass dish on top of a piece of filter paper, and 1 ml sterilized water was added to the paper to maintain the moisture. PDA medium blocks of 5 mm diameter containing actively growing *Fusarium* were inoculated on the surface of each leaf. For each leaf, one mycelium block was inoculated to a wounded site created by a cut with a sterilized razor (top right of leaf) and another block was inoculated to a healthy uncut site (bottom right of leaf). As negative controls, sterile PDA medium blocks without any *Fusarium* mycelia were similarly inoculated to the cut (top left of leaf) and uncut site (bottom left of leaf). The glass dishes containing treated leaves were placed in an artificial climate chamber with a light/dark cycle of 16 h/8 h, and the temperature was set to 25°C and 16°C, respectively in light and dark. The leaves were observed twice a day for symptoms, and the diameters of the lesions were measured and recorded after 96 h of incubation. Fungi from the diseased leaves were further isolated following the procedures described above for isolating the original pathogens from wilted plants. The re-isolated fungi were then compared with the original inoculated to confirm their identity based on morphological features and *ef1*-α sequences.

### SSR Genotyping for FOSC Isolates

Previous studies have developed SSR markers for one of the most common *Fusarium* species, *F. oxysporum* (Bogale et al., 2005; Cruz et al., 2018), now called the *F. oxysporum* species complex (FOSC) (Lombard et al., 2019). Here we selected six highly polymorphic SSR markers as identified in previous studies to investigate the genetic relationships among FOSC isolates obtained from wilted yams. The primer names and sequences are shown in Table 2. For each isolate at each SSR marker, the PCR reaction (15 μL) consisted of 7.5 μL 2 × Tsingke MasterMix, 1 μL of each primer (10 μM), and 1 μL template DNA. PCR cycles consisted of a 5-min denaturation (at 94°C), followed by 30 cycles of denaturation (94°C), annealing (50−55°C) and extension (at 72°C), each for 303s. PCRs were terminated after a final 5-min extension. Successful amplifications of the SSR markers were confirmed by agarose gel electrophoresis before being processed for fragment analyses and allelic identifications described below (Pouralibaba et al., 2018).

**TABLE 2:** Primer sequences and allele series for the six simple sequence repeat (SSR) marker loci analyzed in the collection of 67 FOSC isolates causing yam wilt in South-Central China.

Locus	Primer sequence (5′−3′)	Sizes of SSR alleles (bp) among the 67 isolates	Total number of alleles
FOMSSR-2	TCATTCTCCATGTCCTCATC TCGTTCCGATAGTAATTCGTCA	163,165,167,169,173,175,182,184, 190,192,200	11
FOMSSR-6	ACACTCCAAGAACTCAGCATCA GACAAAACTCGCTATTCGTTCC	198,200,201,202,204,207,209,210, 217,219,226,228,236	13
MB2	TGCTGTGTATGGATGGATGG CATGGTCGATAGCTTGTCTCAG	246,251,252,254,257,258,260,264, 266	9
MB9	TGGCTGGGATACTGTGTAATTG TTAGCTTCAGAGCCCTTTGG	132,134,139,140,141,142,143,156, 166	9
MB13	GGAGGATGAGCTCGATGAAG CTAAGCCTGCTACACCCTCG	227,240,245,252,269,279,281,282,285,286,288,304,307,322,326	15
MB17	ACTGATTCACCGATCCTTGG GCTGGCCTGACTTGTTATCG	311,313,316,317,328,329,330,331,335,341,344	11

For each of these six SSR markers, the forward primer was labeled with fluorophores FAM or HEX (TSINGKE Biological Technology), the PCR product was subjected to secondary amplification. The amplification system and conditions were the same as those of the first amplification. After amplification, 0.5 μL of amplification product was mixed with 10 μL of a mixture containing highly deionized-formamide and rox-500 fluorescent molecular weight internal standard in a ratio of 130:1. The mixtures were then denatured at 95°C for 5 min, place on ice for 10 min, loaded onto an ABI 3730xl automatic DNA analyzer for capillary electrophoresis. The output files were analyzed using the GeneMapper 4.1 software to identify the lengths of amplified fragments of all FOSC isolates (Chatterji and Pachter, 2006).

### Data Analysis

#### Species Diversity

For each region, the frequency of each putative *Fusarium* species in each geographic region was determined by dividing the number of isolates within each species by the total number of the *Fusarium* molds isolated in the regions. In addition, we also calculated the Simpson’s species diversity index using the formula D = (1-ΣP_*i*_^2^); where P_*i*_ is the frequency of isolates of species “i.” The index describes the probability that two random isolates in each sample belong to different species. The statistical significance of *Fusarium* species distribution differences between the regions were determined using a Chi-square test against the null hypothesis that there was no difference between different regions.

#### Population Genetic Variation Within FOSC

Due to the shared SSR markers among species within FOSC and the relatively large number of isolates in this species complex, we conducted the following analyses including all strains within FOSC. The number and frequency of alleles were calculated by software PowerMarker v 3.25 at each locus for each geographic sample (Liu and Muse, 2005) according to the difference in band size of the microsatellite amplification products of different strains. In the second, strain relationships based on microsatellite markers were analyzed using the UPGMA method using MEGA X (Kumar et al., 2016). In the third, the contribution of geographic isolation to the total genetic variation in the FOSC sample was estimated based on the analysis of molecular variance (AMOVA; Excoffier et al., 1992). Fourth, genetic differences between pairs of geographic populations were analyzed based on the traditional *F*_*ST*_ values. Only populations with at least 10 isolates were included for population differentiation analyses. Both the AMOVA and pairwise population F_*ST*_ values were obtained using the GenAlex V 6.5 software, with statistical significance derived from 1000 permutations (Peakall and Smouse, 2012). Lastly, we estimated the putative number of genetic clusters K in this sample using the program STRUCTURE version 2.3.4 (Pritchard et al., 2009). Using the admixture model, 10 replicated runs of *K* = 1−7 were carried out after a burn-in period of 100,000 generations followed by a run length of 1,000,000 generations. The number of genetical clusters (K) was identified by following the method described by Evanno et al. (2005).

#### Allelic Associations Within FOSC

We examined the associations among alleles at the six SSR loci using two methods: phylogenetic compatibility between pairs of loci and the overall index of association (I_*A*_). Both tests were performed using the program Multilocus version 1.3b (Agapow and Burt, 2001). The null hypothesis for I_*A*_ is that frequent recombination occurs and is seen in random associations among alleles at the different loci. Significant deviations from random associations would result in rejection of the null hypothesis and support the alternative hypothesis of linkage, or infrequent or no recombination of the populations in the field. The statistical significance of this test was derived by running 999 randomized permutations of recombined datasets. In contrast, the phylogenetic incompatibility test used strict clonality and no recombination as the null hypothesis. The details of these two tests are described elsewhere (Agapow and Burt, 2001). We examined allelic associations within individual species with sample sizes greater than 10 as well as for the combined FOSC. Recombination is inferred when the value of I_*A*_ is low and there is broad phylogenetic incompatibility between pairs of loci. Evidence for prevalent clonal reproduction is inferred when the I_*A*_ value is high and there is a lack of phylogenetic incompatibility.

## Results

### Isolation and Putative Identification of *Fusarium* Species

**FIGURE 1:**
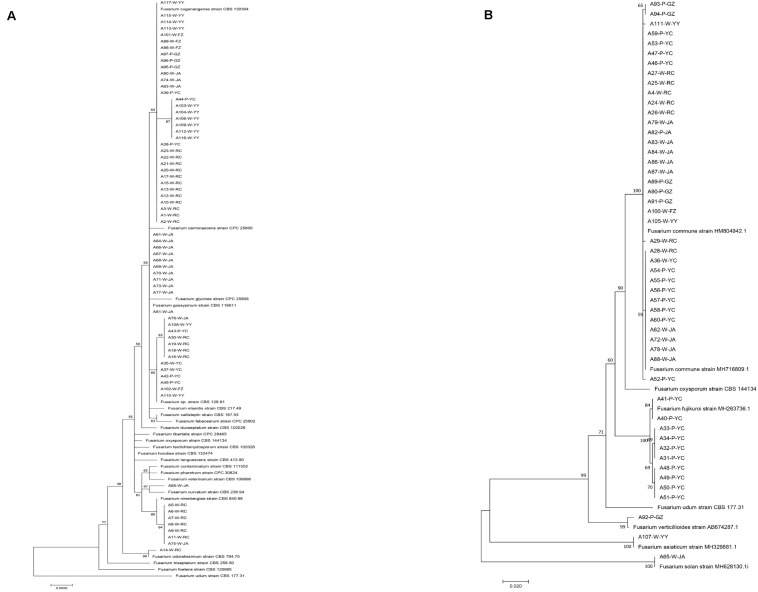
Phylogenetic relationships among our strains and those of the closely related *Fusarium* species based on *ef1*-α nucleotide sequences. **(A)** Relationships among our FOSC isolates and the representative sequences of 23 strains related to our strains. **(B)** Relationships among our remaining 50 *Fusarium* isolates with representative sequences of eight closely related *Fusarium* species. The names of species and type/epitype strains representing those closely related to our strains were from Lombard et al. (2019) and GenBank.

Through isolation and culturing of diseased yam tissue samples collected from six regions in South-Central China, we obtained a total of 117 strains of *Fusarium*, with each isolate from a different diseased tissue piece (Table 1). These isolates came from 37 of the 52 collected diseased plants. Among the 117 strains, 30 were from each of Ruichang and Yichun, 28 from Ji’an, 9 from Ganzhou, 5 from Fuzhou, and 15 from Yiyang. Based on their colony and microscopic morphological characteristics as well as DNA sequences at the *ef1*-α locus, the 117 isolates were found to belong to 11 *Fusarium* species. For several reasons, our species identifications are tentative here. First, several of the closely related species have very similar *ef1*-α sequences (Lombard et al., 2019; Figure 1), making identification based on *ef1*-α sequences alone difficult. Second, there were subtle *ef1*-α sequence variations among our strains within five of the 11 putative species and some of those variant strains may represent novel species. Third, some of our strains and those of the type and epitype strains of the closely related species were not 100% identical, thus potentially they could also represent new taxa. For these reasons, we will use “species affinis” or “aff.sp.” for short to represent the tentative nature of our species identifications. Specifically, of the 11 putative species, six belonged to FOSC (*F. aff. cugenangense, F. aff. curvatum, F. aff. gossypinum, F. aff. nirenbergiae, F. aff. odoratissimum*, and *Fusarium aff.* sp.). The other five species were *F. aff. asiaticum, F. aff. commune*, *F. aff. fujikuroi*, *F. aff. solan*i, and *F. aff. verticillioides* (Table 3). A Maximum Likelihood tree showing the relationships among our strains and those of their known close relatives based on *ef1*-α sequences is shown in Supplementary Figure S1. Because of the large number of strains and the relatively minor differences in DNA sequences between many closely related species, to improve visualization, we split Supplementary Figure S1 into two sub-figures, Figures 1A,B. The phylogenetic tree in Figure 1A includes 67 strains of the FOSC and the representative sequences of 23 strains closely related to them (Lombard et al., 2019). The phylogenetic relationships between the remaining 50 isolates with representative sequences of eight *Fusarium* species from GenBank are shown in Figure 1B. In the phylogenetic tree, the 67 FOSC isolates were divided into six putative species (Figure 1A), while the remaining 50 isolates were clustered with representative isolates of the following five species *F. asiaticum, F. commune*, *F. fujikuroi*, *F. solani*, and *F. verticillioides* (Figure 1B).

**TABLE 3:**
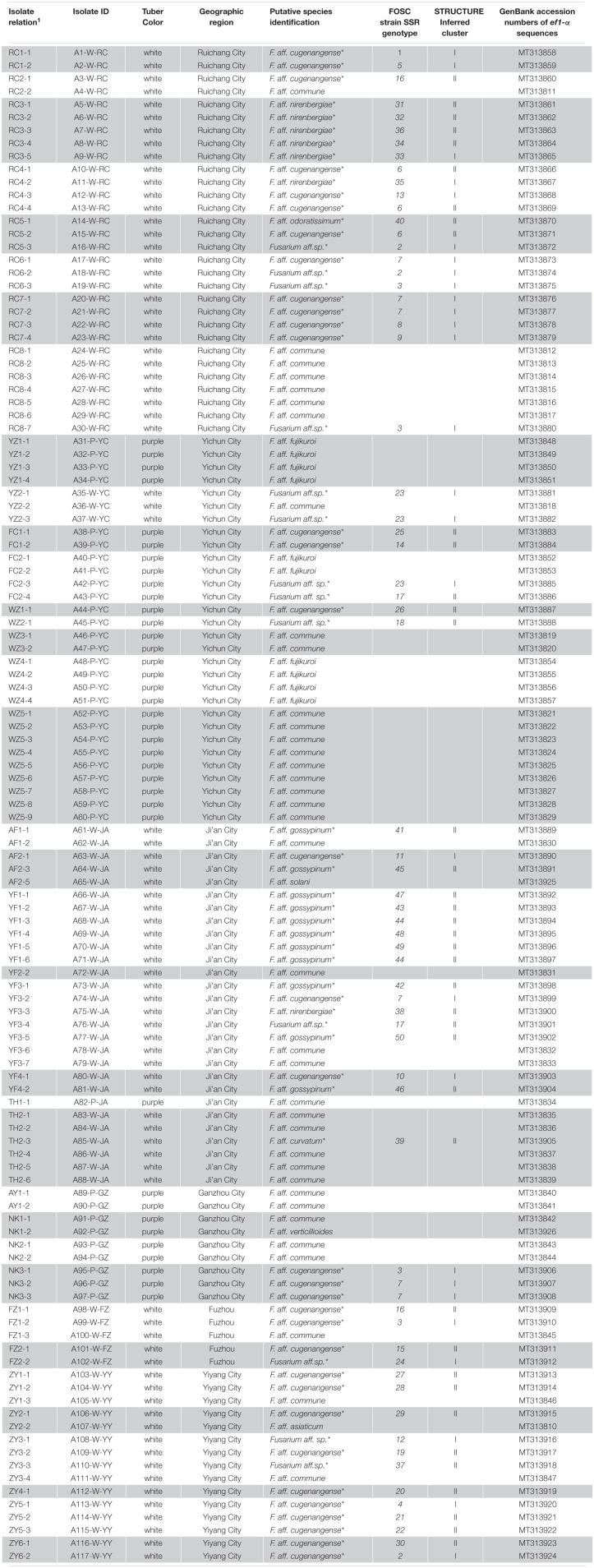
Species and genotype identifications of the 117 *Fusarium* isolates obtained from diseased yam tissues in South-Central China.

Consecutive isolates highlighted in the same color were from different diseased tissues of the same plant. *These species are within the F. oxysporum species complex (FOSC).

Among the 11 putative species, the most common species was *F. aff. commune*, containing 37 strains and representing 31.6% of the total sample. This was sequentially followed by *F. aff. cugenangense* (34 isolates, 29.1%), an undescribed novel species *Fusarium aff.* sp. (13 isolates, 11.1%), *F. aff. gossyypinum* (11 isolates, 9.4%), *F. aff. fujikuroi* (10 isolates, 8.5%), *F. aff. nirenbergiae* (7 isolates, 6%), and one isolate each (0.85%) of *F. aff. solani*, *F. aff. verticillioides*, *F. aff. asiaticum*, *F. aff. curvatum*, and *F. aff. odoratissimum*. Of these 11 putative species, the following six belonged to the FOSC: *F. aff. cugenangense, F. aff. curvatum*, *F. aff. gossyypinum, F. aff. nirenbergiae, F. aff. odoratissimum*, and *Fusarium aff.sp*, accounting for 57.3% (67/117 isolates) of the total *Fusarium* population. Overall, our results show that FOSC and *F. aff. commune* are the dominant species complex/species causing *Fusarium* yam wilt in these six geographic regions (Tables 3, 4).

**TABLE 4:** Information on the species distribution of *Fusarium* strains isolated from yam samples from South-Central China.

*Fusarium* species	Jiangxi					Hunan	
	Ruichang	Yichun	Ji’an	Ganzhou	Fuzhou	Yiyang	Total
FOSC	23(76.7%)	8(26.7%)	17(60.7%)	3(33.3%)	4(80.0%)	12(80.0%)	67
F. aff. cugenangense*	12(40.0%)	3(10.0%)	3(10.7%)	3(33.3%)	3(60%)	10(66.7%)	34
Fusarium aff. sp.*	4(13.3%)	5(16.7%)	1(3.6%)	0	1(20%)	2(13.3%)	13
F. aff. gossypinum*	0	0	11(39.3%)	0	0	0	11
F. aff. nirenbergiae*	6(20.0%)	0	1(3.6%)	0	0	0	7
F. aff. curvatum*	0	0	1(3.6%)	0	0	0	1
F. aff. odoratissimum*	1(3.3%)	0	0	0	0	0	1
*F. aff. commune*	7(23.3%)	12(40.0%)	10(35.7%)	5(55.6%)	1(20%)	2(13.3%)	37
*F. aff. fujikuroi*	0	10(33.3%)	0	0	0	0	10
*F. aff. solani*	0	0	1(3.6%)	0	0	0	1
*F. aff. verticillioides*	0	0	0	1(11.1%)	0	0	1
*F. aff. asiaticum*	0	0	0	0	0	1(6.7%)	1
Total	30	30	28	9	5	15	117
Simpson’s species diversity index	0.73	0.69	0.70	0.57	0.56	0.52	0.78

*, These species belong to the Fusarium oxysporum species complex (FOSC).

### Diversity and Distribution of *Fusarium* Species

Table 4 summarizes the *Fusarium* species distribution among the six geographical regions. The *Fusarium* species richness and Simpson’s species diversity index for different sites within each of the six regions in Jiangxi and Hunan provinces were presented in Tables 1, 4. Within each of the six regions, at least three putative *Fusarium* species were isolated. Specifically, two geographic samples (Ganzhou and Fuzhou) contained three putative *Fusarium* species each; two (Yiyang and Yichun) contained four putative *Fusarium* species each, one (Ruichang) had five putative *Fusarium* species, and one (Ji’an) had seven putative *Fusarium* species. Though species richness among the regions varied by two folds, the diversity indices were highly similar, all above 0.52. The highest species diversity was found in Ruichang (0.73), followed by Ji’an (0.70), Yichun (0.69), Ganzhou (0.57), Fuzhou (0.56), and Yiyang (0.52) (Table 4).

At the individual plant level, we successfully isolated *Fusarium* strains from 37 of the 52 diseased yam plants. Of these 37 plants, three were found to be infected by three or more putative species of *Fusarium*, 15 were found to be infected by two putative species of *Fusarium* (Table 3) while the remaining 19 were found to be infected by one putative species each. Of these 19 diseased plants infected by a single putative *Fusarium* species, eight were infected by *F. aff. cugenangense*, six by *F. aff. commune*, two by *F. aff. fujikuroi*, and the other three were infected by *F. aff. gossypinum, F. aff. nirenbergiae*, and *Fusarium aff.sp.*, respectively (Table 3).

We further determined whether the different *Fusarium* species were distributed differently among the geographic regions. Here, only the following four geographic regions with sample sizes greater than 15 were used for pairwise comparisons: Ruichang, Yichun, Ji’an, and Yiyang. Due to their small sample sizes, the remaining two geographic samples (Ganzhou and Fuzhou) were not included in the analyses. Furthermore, for the Chi-square contingency table test, due to the relatively small sample sizes of individual species and the close relationships among these six species within FOSC, we treated FOSC as one taxonomic group in this test. Our analyses showed that the Yichun population had a significantly different *Fusarium* species composition from other three regions (Chi-square values all >9.5, *df* = 2, *p* < 0.01). However, the *Fusarium* species compositions among the remaining three regions (Ruichang, Ji’an, and Yiyang) were not significantly different from each other (Chi-square values all <3, *df* = 1, *p* > 0.1). Specifically, in Yichun, the most common species was *F. aff. commune* (40%), followed by *F. aff. fujikuroi* (33.3%), with *Fusarium aff.* sp. (16.7%) and *F. aff. cugenangense* (10.0%) being the third and fourth common species. Interestingly, *F. aff. fujikuroi* was only found in Yichun. In three of the remaining five regions (Ruichang, Fuzhou, and Yiyang), the most common species was *F. aff. cugenangense*, followed by *F. aff. commune*. In Ji’an, the most common species was *F. aff. gossypinum*, followed by *F. aff. commune* (Table 4). At present, the sample size from Ganzhou is too small to draw any meaningful conclusion about the prevalence of individual *Fusarium* species and in the comparisons with other regions about *Fusarium* species composition.

The geographic differences in *Fusarium* species distribution prompted us to further investigate the potential relationships between yam variety and *Fusarium* pathogen species distribution. Based on the flesh color of the collected yam tuber samples, the yams are divided into two varieties: white yam and purple yam (Table 3). Among the total of 117 *Fusarium* strains we isolated, 80 were from white yam (68.4%) and the remaining 37 were isolated from purple yam (31.6%). Of the 80 strains from white yams, *F. aff. cugenangense* was the most frequent (28/80, 35.0%), followed by *F. aff. commune* (20/80, 25%), *F. aff. gossypinum* (11/80, 13.8%), *Fusarium aff.* sp. (10/80,12.5%), *F. aff. nirenbergiae* (7/80, 8.8%), with one strain each of *F. aff. asiaticum, F. aff. curvatum, F. aff. odoratissimum*, and *F. aff. solani*. In contrast, of the 37 strains from purple yams, 17 were *F. aff. commune* (45.9%), 10 were *F. aff. fujikuroi* (27%), six were *F. aff. cugenangense* (16.2%), three were *Fusarium aff.* sp. (8.1%) and one was *F. verticillioides*. The difference in *Fusarium* pathogen species distribution between the two yam varieties was statistically significant (Chi-square value = 40.980, *df* = 10, *p* < 0.0001).

### Pathogenicity Tests on Yam Leaves

**FIGURE 2:**
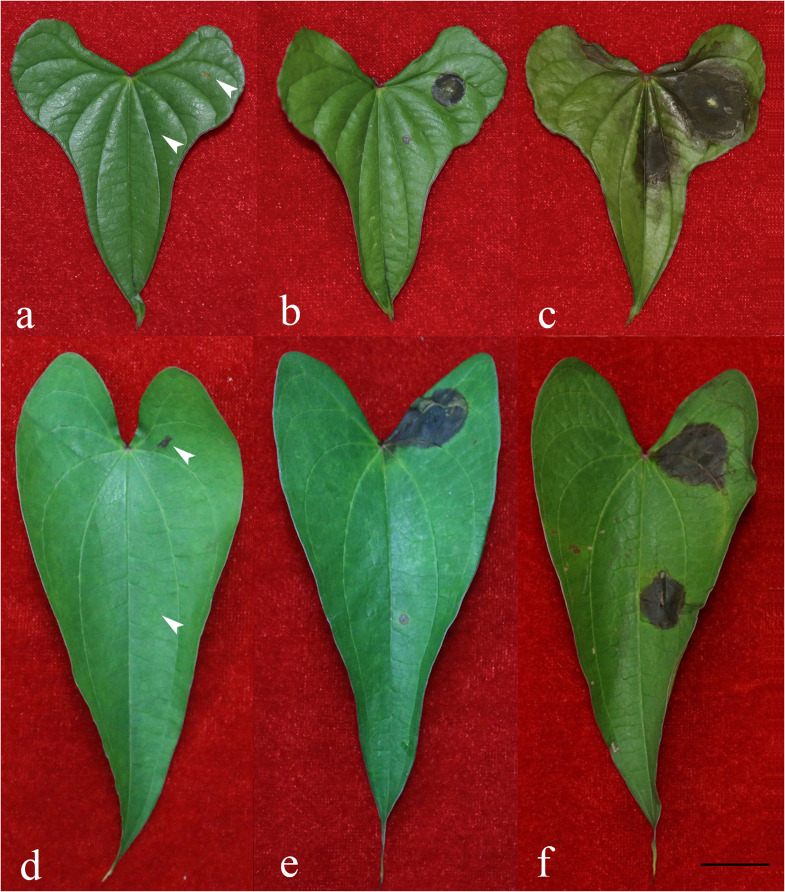
Symptoms on yam leaves inoculated with representative *Fusarium* isolates and negative controls 4 days after inoculation. **(a)** Negative control on white yam leaves; **(b)** *F. aff. commune* strain A62-W-JA; **(c)** *F. aff. cugenangense* strain A63-W-JA; **(d)** negative control on purple yam leaves; **(e)** *F. aff. cugenangense* strain A38-P-YC; **(f)** *F. aff. fujikuroi* strain A40-P-YC. For each leaf, the left side were inoculated two un-colonized PDA medium blocks with one on an injured site created by a cut with a sterile razor (top left) and another block on an intact site without any injury (bottom left). On the right side of each leaf, two PDA medium blocks colonized with *Fusarium* were inoculated, one on an injured site created by a cut with a sterile razor (top right) and another block on an intact site without any injury (bottom right). Scale bar = 2 cm.

A total of eight isolates were tested for their pathogenicity, four isolates were from Ji’an and four from Yichun. After incubation for 2 days, the leaves of yams inoculated with certain *Fusarium* isolates produced a black-brown round spot with a size similar to the inoculated agar block. The spots gradually expanded over the next 2 days, with fungal hyphae appearing in the center of each spot. Figure 2 shows representative pathogenicity test symptoms. In severe cases, the lesion was surrounded by watery stains with signs of fungal spread along the leaf veins (Figures 2c,f). The negative control inoculations and leaves showed no disease symptom (Figures 2a,d). After 4 days, the disease incidence based on visible symptoms for the injured inoculations of both the white yam leaves and purple yam leaves was 100%. For the non-injured inoculations, after 4 days, the disease incidence based on visible symptoms with white yam leaves was 57.8%, and that with purple yam leaves was 19.4%. Below we briefly describe the results of the pathogenicity test.

Among the four isolates from Ji’an representing *F. aff. cugenangense*, *F. aff. commune*, *F. aff. gossypinum*, and *F. aff. solani*, that of *F. aff. cugenangense* (Figure 2c) showed the largest diseased area for both the injured inoculation [mean diameter of diseased tissue = 1.45 cm (SD = +0.11)] and non-injured inoculation [1.171 cm (SD = +0.041)]. Isolate of *F. aff. commune* (Figure 2b) showed the second strongest pathogenicity, with a diameter of injured inoculation at 1.171 cm (SD = +0.072), and that at the non-injured site at 0.871 cm (SD = +0.099). Isolate of *F. aff. gossypinum* had the diameter of injured inoculation of 0.957 cm (SD = +0.012), and that at the non-injured site was 0.300 cm (SD = +0.146). Finally, the diameter of lesion for isolate representing *F. aff. solani* at the cut site was 0.600 (D = 0.061) while the non-injured inoculation had no symptom.

Among the four isolates representing *F. aff. commune, F. aff. cugenangense*, *F. aff. fujikuroi*, *Fusarium aff.* sp. isolated from purple yam from Yichun, the isolate representing *F. aff. cugenangense* (Figure 2e) inoculated at the injured site had a mean diameter of diseased area at 1.979 cm (SD = +0.042), while at the non-injured site was 0.050 cm (SD = +0.008). The *F. aff. fujikuroi* isolate (Figure 2f) caused a similar damage at the injured site as the *F. aff. cugenangense* isolate, with a mean diameter of diseased area at 1.936 cm (SD = +0.059), while that for the non-injured inoculation was 0.193 cm (SD = +0.260). The diameter of a *Fusarium aff.* sp. strain inoculated at the injured site was 1.929 cm (SD = +0.247), and that at the uninjured site was 0.102 cm (SD = +0.033). Finally, the inoculated *F. aff. commune* strain had the diameter of diseased area at 1.657 cm (SD = +0.245), and that at the non-injured site was 0.329 cm (SD = +0.208).

For representative diseased leaves caused by each of the eight inoculated *Fusarium* strains, we re-isolated the fungal pathogen. These re-isolated strains were compared with the original inoculated strains for their colony cultural characters, microscopic features, and *ef1*-α sequences. Our analyses confirmed that those re-isolated fungi from the diseased leaves were identical to those inoculated. Together, these results demonstrated that the *Fusarium* strains isolates here are all capable of causing diseases in their respective yam varieties where they were originally isolated from.

### Genetic Variation and Relationships Among Strains of FOSC

**FIGURE 3:**
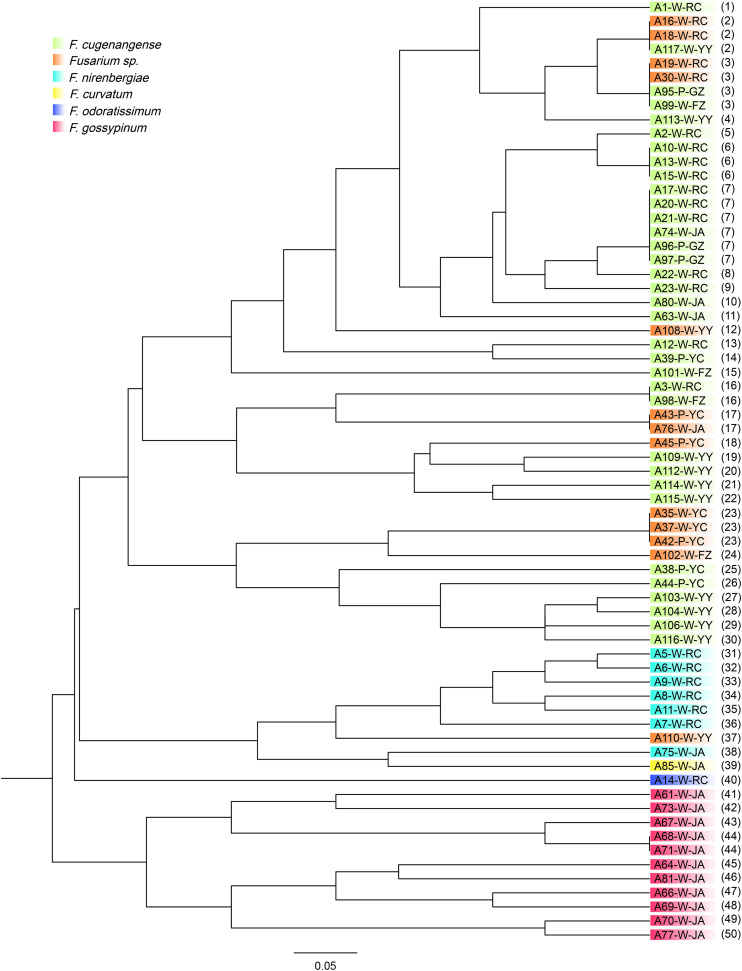
Genetic relationships among 67 strains of FOSC isolated from yam plants with vascular wilt syndrome in six geographic regions in South-Central China. The isolate codes correspond to those in Table 3. W, yams with white tuber; P, yams with purple tuber. The last two letters correspond to their six geographic regions. The numbers in parenthesis refer to their SSR genotypes corresponding to those in Table 3. Different colors represent different species within FOSC identified based on their *ef1*-α nucleotide sequences as detailed in Lombard et al. (2019).

All six microsatellite marker loci were polymorphic in our samples of 67 FOSC strains. Among these six loci, the average number of alleles per SSR locus was 11, ranging from 9 (for marker loci MB2 and MB9) to 15 (for marker locus MB13) (Table 2). The strain relationships based on SSR data are presented in Figure 3. In total, the 67 strains belonged to 50 multilocus microsatellite genotypes (MLMGs) (Figure 3 and Table 3). Eight of the 50 MLMGs were represented by two or more isolates each while the remaining 42 were represented by one isolate each. Of the eight shared MLMGs, only one (MLMG#44) contained isolates exclusively from the same diseased plant. Three other MLMGs (#6, #7, and #23) each contained isolates from different diseased tissues of the same plant, but also contained isolates from other plants from either the same geographic region (MLMG#6 and #23) and/or from different geographic regions (MLMG#7). Of the remaining four shared MLMGs, MLMG#2 contained three isolates from different plants of both the same region and different regions; MLMG#3 contained four isolates from four different diseased plants from three different regions; and MLMGs#16 and #17 contained two isolates each with each of their isolates from a different geographic region. Among the 50 MLMGs, MLMG#7 was the most frequently observed, represented by 6 isolates from five different diseased plants located in four geographic regions (Figure 3). Overall, the patterns of genotype sharing between isolates of FOSC from different geographic regions are consistent with long-distance dispersal of this fungal pathogen in Jiangxi.

Similar to the above-mentioned genotype sharing between strains of FOSC from different geographic regions, several groups of *Fusarium* isolates from the two yam varieties also shared MLMGs. Specifically, MLMGs #3, #7, and #23 contained isolates from both the white and purple varieties of yams, often from different geographic regions. In contrast to the genotype sharing among isolates from different geographic regions and different yam cultivars, isolates of FOSC from different diseased tissues of the same plant often have different MLMGs. Indeed, of the 20 diseased plants where more than one isolates of FOSC were analyzed for each, only one (plant YZ2) contained isolates of the same genotype (MLMG#23) (Table 3). The remaining 19 plants each were infected by two or more MLMGs of FOSC. For example, all five *F. aff. nirenbergiae* isolates from plant RC3 each had a unique MLMG (Table 3). Taken together, the results demonstrate a high genotype diversity of FOSC around most individual diseased plants in most regions.

By comparing the relationships between the species classification based on *ef1-α* sequences and multilocus microsatellite genotypes based on the six SSR markers for the 67 FOSC strains, we found that they were largely consistent with each other (Figures 1, 3). The only species showing inconsistent clustering between the *ef1-α* phylogeny and the SSR genetic relationship tree was the potential novel undescribed species. Isolates of this species were distributed throughout the *F. aff. cugenangense* isolates in SSR phylogram. In addition, one isolate of this putative species was clustered with strains of *F. aff. nirenbergiae.* In contrast, strains of *F. aff. gossypinum* formed a distinct SSR genotype cluster.

### Population Structure of FOSC

At the population level, each of the six geographic populations of FOSC contains a diversity of MLMGs that are distributed across the dendrogram (Figure 3). However, most geographic populations also contain clusters of strains with similar or identical genotypes. For example, 11 strains from Ji’an formed a cluster (MLMGs 41-50; Figure 3 and Table 3). Similarly, six strains from Ruichang (MLMGs #31-36) and four from Yiyang (MLMGs #27-30) were clustered with each other according to their geographic locations (Figure 3 and Table 3).

The overall analysis of molecular variance (AMOVA) revealed that 84.3% of the total observed genetic variation was found within regions while 15.7% (*p* = 0.047) of the variation could be attributed to geographic separation among regions. This result is consistent with a low but statistically significant level of genetic differentiation among geographic populations. Pairwise population F_*ST*_ analyses indicated that the three geographic populations with sample sizes greater than 10 isolates each of FOSC (Ruichang, Ji’an, and Yiyang) were significantly differentiated from each other (pairwise *F*_*ST*_ values range from 0.167 to 0.306, *p*-Values range from 0.015 to 0.001). The results are consistent with certain level of dispersal barrier among these three geographic populations.

**FIGURE 4:**
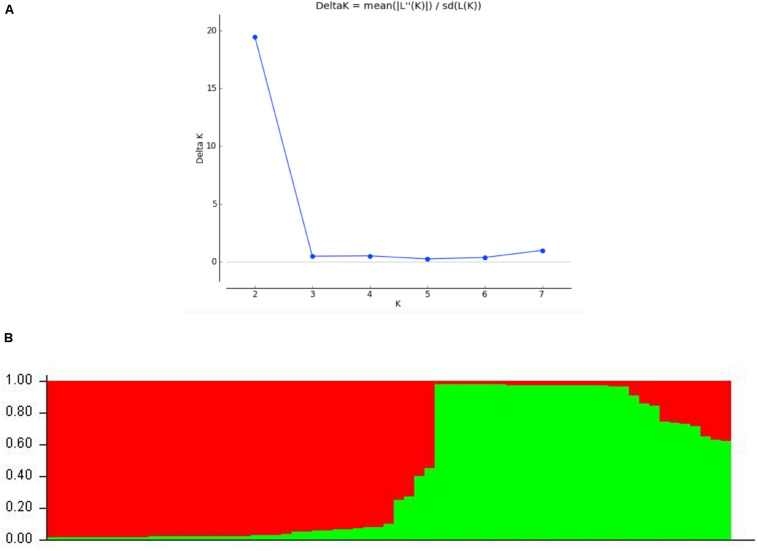
STRUCTURE results of the 67 FOSC isolates based on their genotypes at six simple sequence repeat marker loci. **(A)** Evidence of *K* = 2 as the optimal number of genetic clusters. **(B)** Genetic ancestry association for each of the 67 strains. The red and green represent genetic elements from these two distinct genetic clusters. The placement of individual FOSC strains into either cluster I or II is shown in Table 3.

STRUCTURE analyses showed that the optimal number of genetic clusters was 2, with [LNP (D)] breakpoint appeared in *K* = 2 (LNP (D) = −624.0) (Figure 4A). However, as can be seen from Figure 4B, there is evidence of low but persistent genetic exchanges between these two genetic clusters (I and II). Cluster I contained 29 FOSC and included isolates from three putative *Fusarium* species, including 18 *F. aff. cugenangense* isolates, nine *Fusarium aff.* sp. isolates, and two *F. aff. nirenbergiae* isolates. Cluster II contained 38 isolates belonging to six species of within FOSC, including *F. aff. cugenangense* (16 isolates), *F. aff. gossypinum* (11 isolates), *Fusarium aff.* sp. (4 isolates), *F. aff. nirenbergiae* (5 isolates), and one isolate each of *F. aff. curvatum* and *F. aff. odoratissimum*. While the genetic clusters seem to show large inconsistencies with their species identifications, certain groups of MLMGs shown in Figure 3 belonged to the same genetic cluster. For example, MLMGs #1 to #13 all belonged to genetic cluster I; while MLMGs #36 to #50 all belonged to genetic cluster II.

### Allelic Associations

To assess allelic relationships at the six SSR loci within and between individual species, we obtained the indices of association and phylogenetic compatibility for three putative *Fusarium* species which more than 10 individuals each as well as for the total sample including all 67isolates within FOSC (Table 5). The three individual species were *F. aff. cugenangense* (34 isolates), *Fusarium aff.* sp. (13 isolates), *F. aff. gossyypinum* (11 isolates), and the total sample of FOSC (67 isolates). All four population samples rejected the null hypothesis of random recombination at *p* < 0.001, with the indices of association being 4.45482 for *F. aff. gossyypinum*, 4.38888 for *Fusarium aff.* sp., 1.52086 for FOSC, and 1.42058 for *F. aff. cugenangense*. These results suggest significant clonality within each of the four samples. However, phylogenetic compatibility analyses showed that while the three individual species all showed phylogenetic compatibility, 33.3% for *F. aff. cugenangense*, 86.7% for *F. aff. gossyypinum*, and 93.3% for *Fusarium aff. sp*., incompatibility was found in all three species, rejecting the null hypothesis of strict clonality and consistent with certain levels of recombination within each of the species. Interestingly, the lowest phylogenetic compatibility (6.7%) was found in FOSC, the combined dataset. This result suggests evidence for recombination among the species within FOSC.

**TABLE 5:** Index of association and phylogenetic compatibility among FOSC samples.

Populations	Number of strains	Number of different genotypes	Phylogenetic compatibility (%)	P	Index of association	P
*F. aff. cugenangense*	34	21	33.33	<0.001	1.42058	<0.001
*Fusarium aff.* sp.	13	8	93.33	<0.001	4.38888	<0.001
*F.aff. gossypinum*	11	11	86.67	<0.001	4.45482	<0.001
FOSC	67	46	6.67	<0.001	1.52086	<0.001

## Discussion

In this study, we obtained and analyzed 117 isolates of *Fusarium* from diseased tissues of 37 yam plants. These yam plants were from six geographic regions in two provinces in South-Central China. The putative *Fusarium* species were identified based on DNA sequences at the *ef1-α* locus. In addition, for strains in the most common species FOSC, we obtained their multilocus microsatellite genotypes (MLMGs) at six loci. Our analyses revealed that multiple *Fusarium* species can cause yam wilt. In addition, the same yam plant can be infected by multiple *Fusarium* species and/or multiple genotypes of FOSC. Interestingly, six of the 50 MLMGs were shared by isolates from different geographic regions, suggesting long-distance dispersal. However, significant genetic differentiations were observed between several geographic populations, consistent with the presence of dispersal barriers among the regions. Below we discuss the relevance of our findings to previous studies and the implications for the management and control of *Fusarium* vascular wilt in yams.

Our analyses identified that multiple *Fusarium* species can cause wilt in Chinese yams. Combined with recent taxonomic revisions, our results significantly expand the list of putative *Fusarium* species capable of causing *Fusarium* yam wilt from one in Yao (1998) to the 11 putative species reported here. Interestingly, though most isolates belonged to FOSC, none of our 117 isolates belonged to the newly typified *F. oxysporum* sensu stricto (Lombard et al., 2019; Figure 1). The expanded *Fusarium* species list for yam wilt here is similar to those in several other agricultural crops. For example, six *Fusarium* species have been reported to cause soybean root rot, including *F. avenaceum*, *F. commune, F. equiseti*, *F. graminearum*, *F. oxysporum, F. proliferatum*, and *F. solani*, with *F. oxysporum* showing the strongest pathogenicity (Chang et al., 2018). Similarly, eleven *Fusarium* species were identified from banana fruit rot in Peninsular Malaysia, with *F. proliferatum* being the most virulent (Abd Murad et al., 2017). Other crops such as tomatoes and maize also showed that multiple *Fusarium* species can cause a diversity of diseases in different geographic regions (Akbar et al., 2018; Stępień et al., 2019).

Similar to those in other crops, the exact reasons for the increased number of *Fusarium* species as causal agents of yam wilt are not known. However, three factors could have contributed to the observations. The first is the development of molecular tools and databases that are allowing accurate differentiation of closely related species in *Fusarium* (O’Donnell et al., 2015). For example, in 2003, *F. commune* was separated from *F. oxysporum* and became an independent species according to morphological and molecular phylogenetic data (Skovgaard et al., 2003). In 2019, 15 cryptic taxa within FOCS were proposed and with several new ones still to be described (Lombard et al., 2019). The second reason is the increased sampling for pathogens from multiple diseased tissues of the same plant, from multiple plants in the same geographic region and from different geographic regions. For example, five of the 11 species identified here were only represented by one isolate each (<1% for each of the three species) and they could have been easily missed if fewer plants or tissue samples were taken for analyses (Huang et al., 2013; Zhao et al., 2013). Conversely, if more diseased plants were analyzed, potentially more *Fusarium* species could be found to cause yam wilt disease. The third reason may be related to the increasing host shifts of *Fusarium* pathogens from other host plants to yams. These three possibilities are not mutually exclusive and all three could have contributed to the increased richness and diversity of *Fusarium* species causing yam vascular wilt.

Our results revealed that FOSC was the overall most common species complex causing yam *Fusarium* wilt, accounting for 57% of all the isolates. This result is largely consistent with previous studies where *F. oxysporum* was the only or main species reported as causal agent of yam *Fusarium* wilt in China (Huang et al., 2013; Zhao et al., 2013). Interestingly, our analyses identified that in certain geographic regions such as Yichun and Ganzhou, FOSC was not the dominant pathogen. In Yichun, the purple yam wilt was often more frequently caused by *F. commune* and *F. fujikuroi.* However, we would like to note that both Yichun and Ganzhou mainly grew the purple yam cultivar while the other four regions grew almost exclusively the white yam cultivar. Regardless, these results suggest the potential for geographic and/or host cultivar - based species distributions causing *Fusarium* yam wilt. Our limited pathogenicity tests using yam leaves as model materials confirmed that all the eight tested isolates were capable of causing diseases to yam leaves belonging to the yam variety where they were initially isolated. However, in order to understand the differential prevalence of *Fusarium* species on the two yam varieties (i.e., white and purple tubers), cross-infection experiments are needed to determine whether there is host cultivar-based specificity among the identified *Fusarium* species and genotypes.

Our analyses revealed certain degree of consistency between the species identified based on *ef1-α* sequences and their microsatellite genotypes at six loci. For example, our SSR population genetic data are supportive *F. aff. gossypinum* as a new species within FOSC, consistent with the genotypic species concept (Xu, 2020). However, strains of *F. aff. cugenangense* and the proposed but yet-to-be-described species *F. aff.* sp. as determined using their *ef1-α* sequences were mixed together on the phylogram (Figure 3). Indeed, some of the strains from these two species shared identical SSR genotypes. This result suggests that these two putative species are not reproductively isolated in nature. Furthermore, both the allelic association analyses and the STRUCTURE analyses indicated allelic sharing at the six SSR loci and evidence for genetic exchanges at the population level among the species within FOSC. Thus, caution is needed in calling different genotypes as different species and more evidence should be gathered, preferably based on whole genome sequences, before further division of the FOSC into additional new species are proposed (Xu et al., 2016; Xu, 2020).

Based on allelic information at the six SSR loci, our analyses identified both local and long-distance genotype sharing. Including more SSR markers or other markers may separate isolates of the same SSR genotype based on the six markers into more genotypes. However, using the *ef1-α* sequences and these six SSR markers, we were able to identify multiple species as well as multiple genotypes of FOSC associated the same diseased host plant (Table 3), consistent with the high discriminatory power of these six SSR markers. Since *Fusarium* species are soil-borne, our results suggest that the species diversity and genotype diversity of *Fusarium* pathogens must be very high in many of the yam fields in Jiangxi and Hunan provinces. These yam fields have been used to grow crops by local farmers for centuries. In southern China, farmers typically rotate their crops and vegetables for their fields. However, over the last two decades, due to the relatively high yield of yam tubers and increasing consumer demands, there were decreased crop rotations and increased continuous cropping for yams in the same fields (Liu and Li, 2010). This practice has likely enriched both the species diversity and genotype diversity of *Fusarium* pathogens capable of causing yam wilt. Indeed, a previous study showed that the proportion of *Fusarium* in rhizosphere soil microorganisms from fields with yam wilt diseases was significantly higher than that without wilt diseases (Kang et al., 2017). However, carefully designed experiments to obtain detailed quantitative data are needed in order to understand the relationships among the frequency of crop rotation, the severity of yam vascular wilt, and the diversity of *Fusarium* species and genotypes in these fields.

Our analysis of molecular variance (AMOVA) identified low but statistically significant geographic contribution to the total genetic variation among the six regional populations of FOSC. The pairwise population comparisons of the three geographic populations with relatively large sample sizes also revealed statistically significant genetic differentiations. The statistically significant differentiation contrasts the observation that several genotypes were shared by strains from distinct geographic regions separated by hundreds of kilometers. Taken together, the results suggest that regional populations of FOSC likely each has its own endemic elements. However, long-distance dispersal of asexual spores by natural forces such as wind and by anthropogenic factors such as human travel and yam trade could bring pathogens from one region to another. Similar kinds of genotype sharing have been reported for other plant fungal pathogens and human fungal pathogens, including those in southern China (e.g., Li et al., 2016; Wu J. Y. et al., 2019).

Yam is an important economic crop with significance in both traditional Chinese medicine and food in China. However, in recent years, there has been continued increase in infectious diseases on yams, with vascular wilt being one of the most serious (Sun et al., 2012; Zhu et al., 2016). Our analyses demonstrated that fungi in the genus *Fusarium* represent a common cause of yam wilt, with an overall isolation rate over 70% in Jiangxi and Hunan provinces. The identified *Fusarium* species and genotype diversities and distributions have implications for germplasm conservation and the breeding and cultivation of yams in South-Central China. For example, the differential distribution of *Fusarium* species among regions and yam cultivars suggest that different yam varieties may have different susceptibilities to *Fusarium* infections. As such, diverse genetic resources of yam from different regions should be conserved and systematically screened for breeding purposes. At the practical level, crop rotations should be practiced more often between yam and other crops as well as potentially between different yam cultivars in an effort to reduce the burden of these *Fusarium* pathogens in the soil.

## Data Availability Statement

The datasets presented in this study can be found in online repositories. The names of the repository/repositories and accession number(s) can be found below: https://www.ncbi.nlm.nih.gov/genbank/, MT313810 to MT313926. Six representative strains have been deposited in the Agriculture Culture Collection Center (ACCC) with accession numbers ACCC39696 to ACCC39701.

## Author Contributions

GC designed the research. LX, FD, and HY performed the research. CX, YW, and GC provided the materials. FD, JX, and GC analyzed the data and prepared the manuscript. CY, CJ, LZ, GL, WT, GC, and JX contributed to the literature search, reviewing, and finalizing the manuscript. All authors have read and approved the final manuscript.

## Conflict of Interest

The authors declare that the research was conducted in the absence of any commercial or financial relationships that could be construed as a potential conflict of interest.

## Supplementary Material

FIGURE S1 Phylogenetic relationships among our 117 strains and those of the closely related *Fusarium* species based on *ef1*-α nucleotide sequences.
