# Supplementary figures and images for: Fusarium Species and Fusarium oxysporum Species Complex Genotypes Associated With Yam Wilt in South-Central China
Source: Front Microbiol. 2020 Aug 17;11:1964. doi: 10.3389/fmicb.2020.01964 (PMC7461894; doi:10.3389/fmicb.2020.01964)

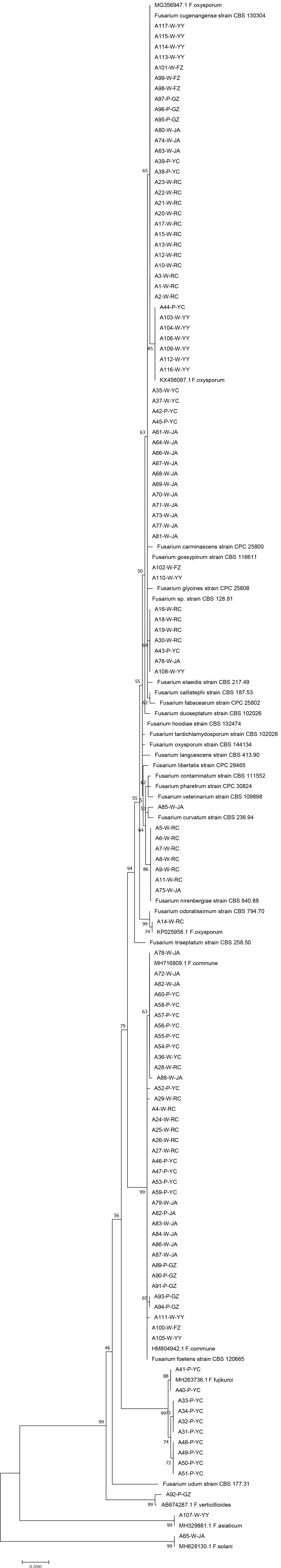

Supplement: FIGURE S1 — Phylogenetic relationships among our 117 strains and those of the closely related Fusarium species based on ef1-α nucleotide sequences. [file Image_1.JPEG]
